# A case of successful treatment of Fournier's gangrene through conservative management and elective debridement

**DOI:** 10.1002/iju5.12706

**Published:** 2024-02-20

**Authors:** Toshifumi Takahashi, Kouhei Maruno, Tatsuya Hazama, Yuya Yamada, Masakazu Nakashima, Kazuro Kikkawa, Masahiro Tamaki, Noriyuki Ito

**Affiliations:** ^1^ Department of Urology Japanese Red Cross Wakayama Medical Center Wakayama Japan

**Keywords:** elective debridement, Fournier's gangrene, Necrotizing fasciitis

## Abstract

**Introduction:**

Fournier's gangrene refers to a necrotizing fasciitis that mainly affects the perineal region and a condition that requires immediate debridement. This case involved elective debridement of Fournier's gangrene after the general condition was improved through antibiotic treatment instead of requesting an emergency debridement.

**Case presentation:**

The patient was an 85‐year‐old man with a performance status of 4 admitted to a nursing home. He was transferred by ambulance with a fever. Blood tests showed a markedly elevated inflammatory response, and computed tomography revealed widespread aerodermectasia around the right testis to the lower abdomen. The patient was diagnosed with Fournier's gangrene. However, his family declined emergency surgical debridement. The patient's general condition was improved with antibiotics, and debridement was eventually performed. After 52 days of hospitalization, the patient was transferred to another hospital.

**Conclusion:**

This study describes the successful treatment of Fournier's gangrene through conservative treatment followed by elective debridement.

**Abbreviations & Acronyms**
ACCI: age‐adjusted Charlson comobidity index
BMI: body mass index
BT: body temperature
CMZ: Cefmetazole
CRP: C‐reactive protein
div.: drip infusion in vein
EF: ejection fraction
FGSI: Fournier's gangrene severity index
FOM: Fosfomycin
GCS: Glasgow Coma Scale
LVFX: Levofloxacin
MEPM: Meropenem
NCCT: noncontrast enhanced computer tomography
p.o.: per os
PS: performance status
qSOFA: quick sequential organ failure assessment score
SFGSI: Simplified Fournier's gangrene severity index
SOFA: sequential organ failure assessment score
UFGSI: Uldag Fournier's gangrene severity index
WBC: white blood cell

**Keynote message**

In cases with a high risk of fatal postoperative complications are ineligible for early surgical treatment, a possible treatment option is elective debridement following conservative treatment, which can possibly reduce the risk of complications for Fournier's gangrene.

## Introduction

Fournier's gangrene refers to a necrotizing fasciitis that mainly affects the perineal region; this condition is often fatal despite the development of corresponding antibiotics.^1^ Immediate debridement is performed on patients who require rescue. Notably, a limited number of reports have discussed elective debridement after antibiotic treatment.

Here, we report a case of elective debridement performed (instead of emergency debridement) after the improvement of the patient's general condition regardless of prior antibiotic treatment.

## Case presentation

**Fig. 1:**
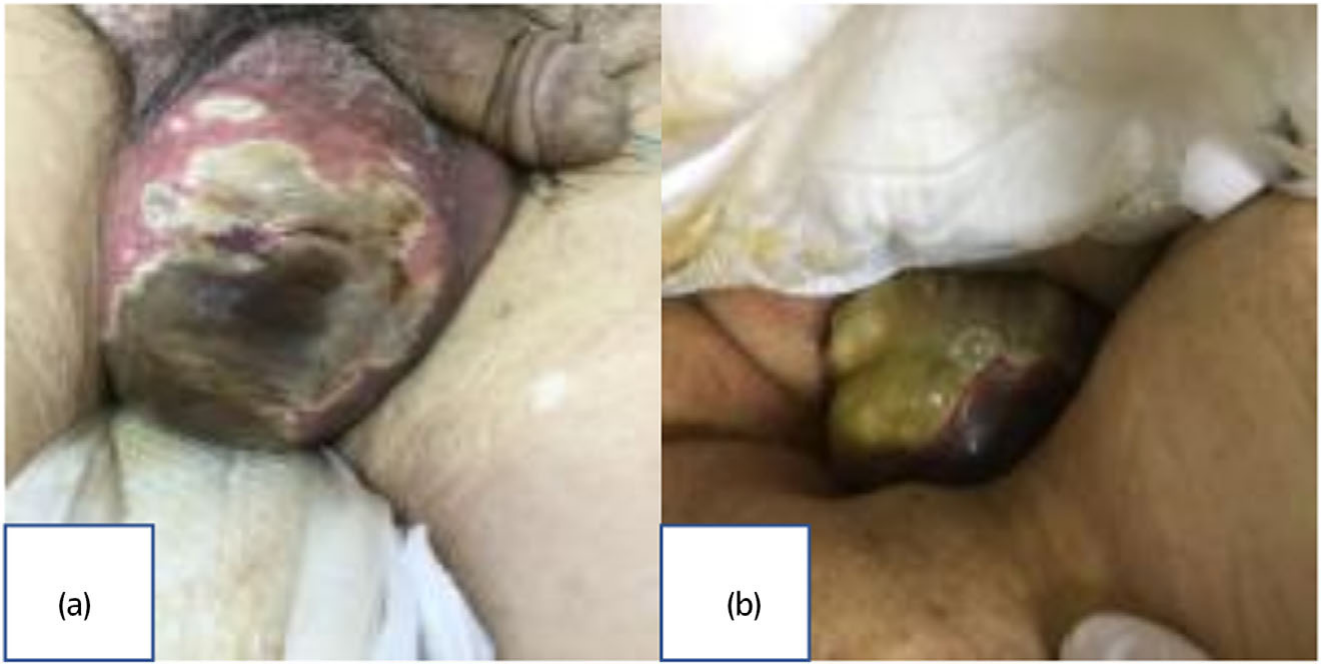
(a) Swelling of the scrotum and partial change of the scrotal skin to black. (b) Swelling of the scrotum and wet skin, but no obvious destruction.

**Fig. 2:**
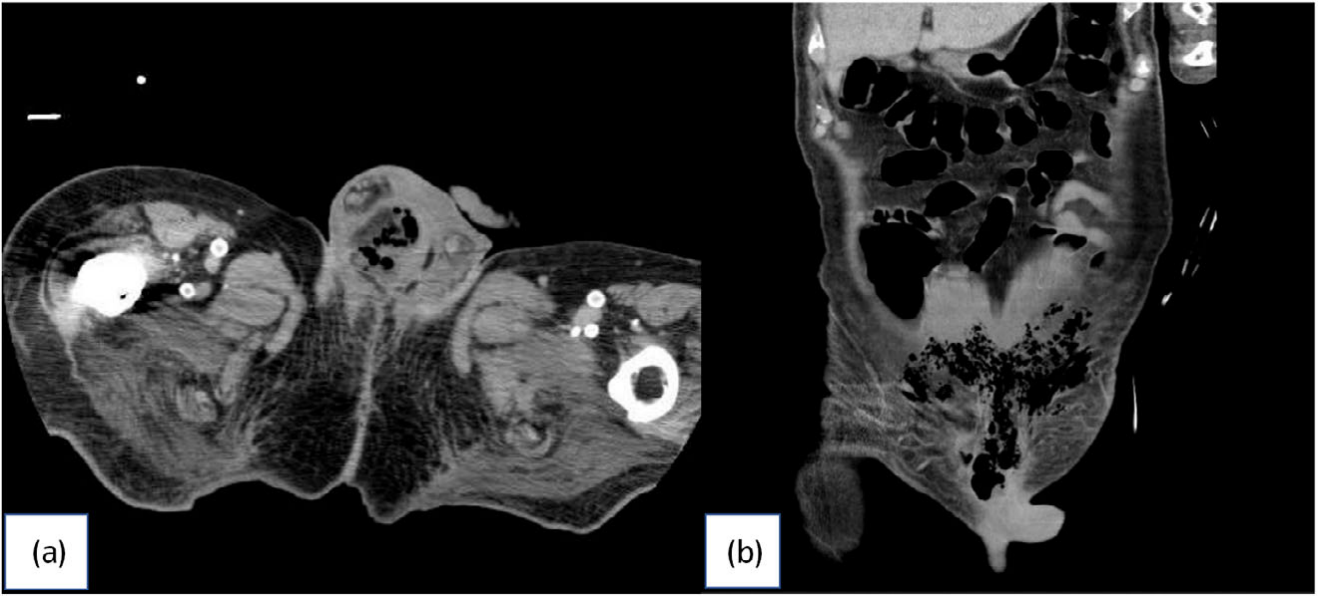
(a) Aerodermectasia around the right testis. (b) Wide spread aerodermectasia around the lower abdomen.

An 85‐year‐old man with a PS4 was admitted to a nursing home. His condition included a fever lasting 2 weeks and oliguria and scrotal pain on the day, which prompted the facility staff to request emergency treatment. The patient was reported to have a medical history of cerebral infarction, heart failure, diabetes, and neurogenic bladder with urethral catheterization. Upon arrival at our hospital, he exhibited a GCS score of 12, and his temperature was 36.5°C. The patient also had a blood pressure of 142/83 mmHg, heart rate of 85 beats/min, respiratory rate of 18 breaths/min, and oxygen saturation of 97% while receiving 0.5% oxygen. Physical examination revealed swelling and tenderness of the scrotum, partial blackening of the scrotal skin (Fig. 1a), and snow grasping sense to the lower abdomen. Echocardiography showed an EF of 30–40%. Blood tests unveiled the following: 21 600 WBC/μL, 26.85 CRP mg/dL, 7.9 g hemoglobin/dL, 1.7 g albumin/dL, 127 mEq sodium/L, and 5.3 mEq potassium/L, which indicate high inflammatory response, anemia, undernutrition, and electrolyte abnormalities. Urine examination revealed pyuria, and NCCT scan showed widespread aerodermectasia around the right testis to the lower abdomen (Fig. 2). Fournier gangrene was diagnosed based on these findings. Systolic blood pressure dropped to the 80s during the test. We informed the patient's daughter regarding the need to perform debridement, intensive care management, and mechanical ventilation, and the difficulty in saving her father with conservative treatment. However, she declined performing invasive procedures including debridement. MEPM, a broad‐spectrum antibacterial agent, was administered to treat the infection by anaerobic bacteria.

**Fig. 3:**
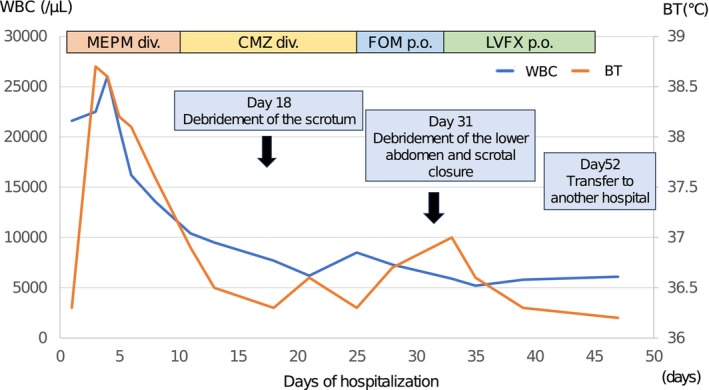
The clinical course after hospitalization.

**Fig. 4:**
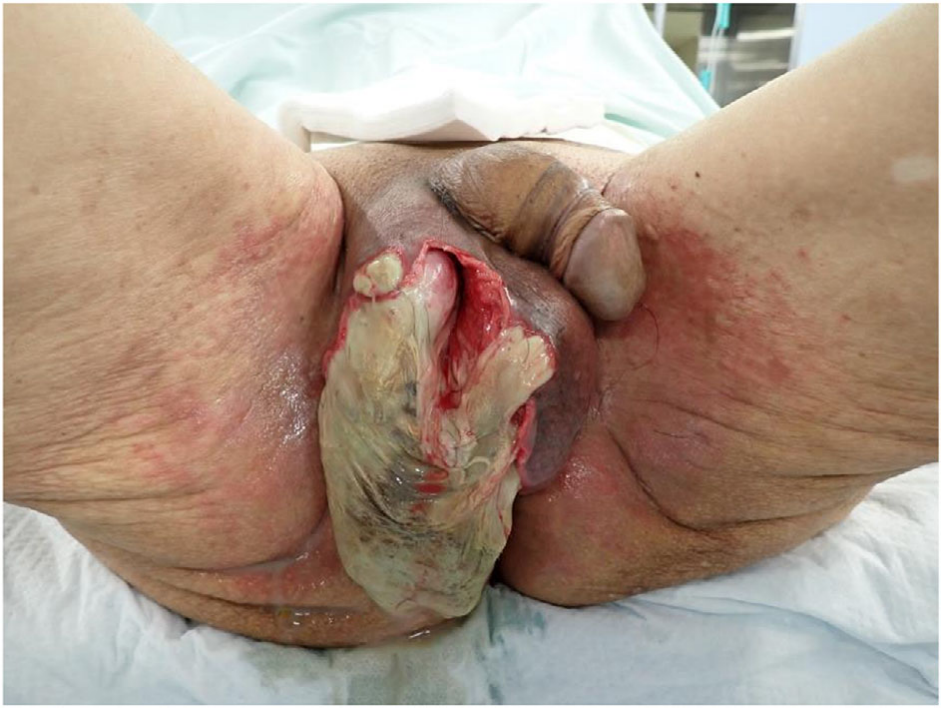
The scrotum had destructed and the right testis was exposed.

The clinical course after hospitalization is shown in Figure 3. After admission, the patient developed a fever of 38.8°C and showed elevated WBC and his consciousness aggravated on the third day. However, from the fourth day, signs of improvement, with a tendency toward the resolution of fever and a decline in WBC, along with the patient's consciousness level, were observed. On the 13th day, the patient experienced swelling of the scrotum and wet skin, but no evident destruction was observed (Fig. 1b). After consultation with the patient's family, the patient underwent debridement of the scrotum on the 18th day. The procedure revealed a necrotic scrotum and exposure of the right testis (Fig. 4). Excision of the necrotic tissue was performed, and the affected area was repeatedly irrigated. Postoperatively observation revealed no fever nor increased inflammatory response, and the patient underwent daily bedside irrigation of the wound and debridement of necrotic tissue. Wound culture revealed the presence of *Enterococcus faecium*, *Enterobacter cloacae*, and *Pseudomonas aeruginosa* (Supplementary Table S1). Because Enterococcus faecium was resistant but in low amounts, oral administration of LVFX was changed from the 28th day. NCCT showed residual aerodermectasia in the lower abdomen. Thus, debridement of the lower abdomen and scrotal closure were performed on the 31st day. The abscess of the lower abdomen was removed, and the organ was repeatedly irrigated. A closed suction drain was installed at the right scrotum, from the lower abdomen through the right inguinal canal, and wound closure was performed. A favorable general condition was observed in the patient postoperatively, which was followed by his transfer to another hospital on the 52nd day. The patient returned to the original facility without major problems 1 month after the transfer.

## Discussion

A type of infectious necrotizing fasciitis, Fournier's gangrene, commonly affects the perineal area and spreads rapidly. The standard treatment for this condition generally includes the immediate surgical removal of the necrotic tissue and administration of antibiotics.^1^ However, regardless of the advancements in emergency medical care, the mortality rate remains high at approximately 20%.^1^ Several risk factors increase the likelihood of death from Fournier's gangrene, and they include heart failure, advanced age, kidney dysfunction, abnormal blood clotting, smoking, alcoholism, low BMI, and diabetes, which are common conditions underlying Fournier's gangrene.^2^

The FGSI and UFGSI scores are useful prognostic predictors of Fournier's gangrene. FGSI is scored using blood test results and vital signs, and UFGSI considers the extent of lesions and age of the patient. An FGSI score over 9 is associated with a 75% mortality rate, and a UGFSI score over 9 is associated with a 94% of mortality rate.^3^, ^4^ The patient had an FGSI score of 7 and a UFGSI score as high as 10 points. In addition, the patient experienced heart failure and low blood pressure, which may be difficult to treat. SFGSI, a simple scoring system, SOFA and quick SOFA, which are used to diagnose sepsis, and ACCI, which is used to predict prognosis, are also useful prognostic predictors of Fournier's gangrene.^5^, ^6^, ^7^

Delayed surgical debridement of necrotizing fasciitis reduces survival rates.^8^ However, elderly people are likely to develop major organ dysfunction, comorbidities, and malnutrition, leading to postoperative complications.^9^ In addition, pneumonia is a frequent postoperative infection and can be fatal to this population.^10^ Periorbital necrotizing fasciitis is often localized and has good blood flow, and conservative treatment with antibiotics has been reported to be successful.^11^ However, except for the periorbital area, there are few reports of the initial conservative treatment of necrotizing fasciitis and subsequent elective surgical drainage. However, in this case, the patient was elderly with PS4 and in poor general condition, and he underwent conservative treatment following his family's request. As the population ages, the number of elderly patients with Fournier's gangrene and poor general condition, who are apt to decline surgical treatment, is expected to increase as observed in this case. Early debridement of Fournier's gangrene is necessary to minimize the risk of fatal postoperative complications. However, in cases where surgery is refused or carries a high risk, conservative treatment with broad‐spectrum antibiotics can be used to improve the patient's condition. Elective debridement can be performed afterward to further reduce the risk of complications.

## Conclusion

This study reported a case of Fournier's gangrene treated via elective debridement after the improvement of the patient's general condition through conservative treatment.

## Author contributions

Toshifumi Takahashi: Conceptualization; data curation; formal analysis; investigation; project administration; writing – original draft. Kouhei Maruno: Data curation. Tatsuya Hazama: Data curation. Yuya Yamada: Data curation. Masakazu Nakashima: Data curation. Kazuro Kikkawa: Data curation. Masahiro Tamaki: Supervision; validation. Noriyuki Ito: Supervision.

## Conflict of interest

The authors declare no conflict of interest.

## Approval of the research protocol by an Institutional Reviewer Board

Not applicable.

## Informed consent

Written informed consent to participate in this study and for the publication of this report was obtained from the patient for ethics approval.

## Registry and the Registration No. of the study/trial

Not applicable.

## Supporting information


**Table S1.** Bacterial count and antibiotics sensitivity of wound culture.
