# Supplementary material for: A case of successful treatment of Fournier's gangrene through conservative management and elective debridement
Source: IJU Case Rep. 2024 Feb 20;7(3):217–20. doi: 10.1002/iju5.12706 (PMC11056266; doi:10.1002/iju5.12706)
Supplement: Supplementary file 1 — Table S1. Bacterial count and antibiotics sensitivity of wound culture. [file IJU5-7-217-s001.docx]

|  | *Enterococcus faecium* | *Pseudomonas aeruginosa* | *Enterobacter cloacae* |
| --- | --- | --- | --- |
| Bacterial counts (cfu/mL) | Low | 2+ | 1+ |
| Susceptibility to  antimicrobial agents |  |  |  |
| Penicillin G | R |  |  |
| Sulbactam / Ampicillin |  |  | R |
| Tazobactam / Piperacillin |  | S | S |
| Cefazoline |  |  | R |
| Cefotiam |  |  | R |
| Cefepim |  | S | S |
| Cefmetazole |  |  | R |
| Meropenem |  | S | S |
| Gentamicin |  | S | S |
| Minocycline | R |  | S |
| Levofloxacin | R | S | S |
| Trimethoprim /Sufamethoxazole |  |  | S |
| Fosfomycin |  |  | R |
| Teicoplanin | S |  |  |
| Vancomycin | S |  |  |

Supplementary table 1 Bacterial count and antibiotics sensitivity of wound culture. R: Resistant, S: Susceptible.
